# Surgical management and outcome of spinal alveolar soft part sarcoma (ASPA): a case series of five patients and literature review

**DOI:** 10.1186/s12957-017-1112-9

**Published:** 2017-02-06

**Authors:** Chenglong Zhao, Xin Gao, Jian Yang, Zhenxi Li, Xiaopan Cai, Tao Tan, Tianhui Hou, Wangjun Yan, Xinghai Yang, Cheng Yang, Tielong Liu, Jianru Xiao

**Affiliations:** 1 Spine Tumor Center, Department of orthopedic oncology, Changzheng Hospital, Second Military Medical University, Huangpu District, Fengyang Rd 415#, Shanghai, China; 2 College of Physical Education and Health, East China Normal University, Minhang District, Dongchuan Rd 500#, Shanghai, China

**Keywords:** Spine, ASPS, Surgical management, Prognostic factors

## Abstract

**Background:**

Alveolar soft part sarcoma (ASPS) is a rare entity of soft tissue malignancies with uncommon spinal involvements. Surgical management should be the best choice of cure.

**Methods:**

Five patients with spinal ASPS were interviewed retrospectively, where data was collected. The relevant literatures were also systematically examined. Thereafter, patient and surgical data were obtained and pooled for prognostic analysis.

**Results:**

A total of five patients with eight surgeries were reviewed retrospectively, and three patients previously reported were also included. All patients were surgically treated, where five of them underwent additional adjuvant therapies such as chemotherapy, radiotherapy, and targeted therapy in order to manage their local and/or systematic diseases. One patient was lost in follow-up. For the remaining seven patients, the mean follow-up period was 19.7 ± 8.8 months, two succumbed to disease while five were alive at the time of the study.

**Conclusions:**

Surgical management is shown to be the most important and the most effective treatment strategy for spinal ASPS, whereas adjuvant therapies made little impact. The prognostic factors for spinal ASPS are primary or metastatic lesions, neurological status, disease progression, systematic conditions, and resection approaches.

## Background

Alveolar soft part sarcoma (ASPS), a rare entity of soft tissue malignancies, predominantly affects young adolescents and accounts for approximately 0.4–1.0% of all soft tissue sarcomas [1, 2]. ASPS is first described by Christopherson et al [3] in 1952 as a unique kind of tumors with uncertain histogenesis. It is characterized by pseudoalveolar, or organoid, arrangement of cells in relation to numerous delicate endothelial-lined vascular channels and septa. ASPS is still classified as “tumors of uncertain differentiation” by the WHO today [4].

ASPS is a tumor with relatively indolent growth pattern, which commonly originates from the muscles and deep soft tissues of the trunk and the extremities; however, metastases may be present at the time of presentation or occur decades after the primary tumor resection [5–8]. The most effective treatment for ASPS is surgical excision while en-bloc resection offers the best chance of cure. Nevertheless, ASPS was previously reported to be refractory to chemotherapy and radiotherapy [9]. Both primary and metastatic spinal ASPS are rarely reported in the literature. There are only sporadic case reports published, which focus on the diagnosis and management [9–12]. In this case series, a retrospective review was performed to report the preliminary experiences of the clinical features and treatment strategies of spinal ASPS, the current literatures are also systematically reviewed.

## Methods

### Patients review

This report retrospectively reviewed patients that were surgically treated and confirmed as spinal ASPS postoperatively in our institution from January 2005 to December 2014. Patient and surgical data such as general information (age and sex), radiological findings, pre- and post-operative status, treatment strategies, operation details, complications, and pathological findings were collected. Lesion classification were classified as primary, locally recurred (surgically treated before), or metastatic. Other organ involvements and metastasis status were also documented. Operation details including the time of surgery, intraoperative blood loss, and reconstruction strategies were recorded. Treatment strategies include surgical management and adjuvant therapies such as chemotherapy, radiotherapy, and targeted therapy. The preoperative and 1-month postoperative Frankel score were calculated and compared for each patient. All patients were followed up after the surgery (every 3 months in the first year and 6 months thereafter), and the progression of their diseases were documented (local recurrence, metastasis, or none). At the last follow-up, the condition of each patient was confirmed by telephone calls and classified as no evidence of disease (NED), alive with disease (AWD), and died of disease (DOD). Permission from the hospital ethics committee and written informed consents from all patients were obtained before the beginning of the study.

### Literature review

The sporadic case reports on spinal ASPS are treasured as valuable resources because the disease type being discussed and explored here is extremely rare with a very low morbidity. A PubMed search was conducted with the combination of the “alveolar soft part sarcoma and spine” since 2000 using the advanced search builder. Three articles were found and all included in the current study. Two independent researchers performed extended literature analysis fulfilling the requirement of this retrospective review. Detailed data were obtained and were pooled together with our clinical data; subsequent prognostic analyses were also performed.

## Results

### General description

Five patients underwent a total of eight surgeries were reviewed retrospectively, and three patients previously reported in the literatures were also included (Table 1). These eight patients have a mean age of 28.4 ± 8.7 years, with a female to male ratio of 5:3. There is a total of four sacrum and seven mobile spine lesions, symptoms include pain, numbness and weakness of the extremities, and/or palpable mass, which were later surgically resected. Other metastatic sites include the lung, the scull, and the pubis. The primary site of tumors was the sacrum for four patients, the thoracic spine for one patient, and the leg for one patient (the primary site of patient no.8 was not available).

**Table 1:** Clinical data of patients with spinal ASPS and literature review

No.	Age, sex	Preoperative status					Treatment					Follow-up			
		Loca	OML	Pri/LR/meta (PS)	AT	F-S pre	Blood loss (ml)	Surgery time (min)	Type	Appro	Comp	F-S post	Follow-up month	LR/meta	Last status
1	28, M	L5	Lung pubis	Meta (right leg)	Targ/radio	C	3200	110	Piecemeal	Post		D	34		AWD
2	30, M	S3-5		Pri		D	1000	230	Piecemeal	Post		E	15		NED
3	22, F	S2	Lung	LR	Chemo	D	NA	230	Piecemeal	Post		E	13		NED
4	44, F	L2-4	Lung, scull	Meta (sacrum)	Chemo/radio	D	1000	155	Piecemeal	Post		D	3	Recu	
		L2/3	Lung, scull	LR	Chemo/radio	C	1000	130	Piecemeal	Post		D	21		AWD
5	16, F	S1-5		Pri		D-C	5000	505	Piecemeal	Post	Wound disunion	D	3	Meta	
		C7T1	Lung	Meta	Chemo	C	2000	170	Piecemeal	Ant		C	1	Recu	
		C6-7	Lung	LR	Chemo	A	1600	300	Piecemeal	Post		B	4		DOD
Zhu et al. [10]	23, M	T12		Pri		NA	NA	NA	Piecemeal	Post	NA	NA	NA	NA	NA
Zadnik et al. [9]	28, F	S1-3		Pri		C	NA	NA	En-bloc	NA	NA	D	26	Recu	DOD
Lizzati et al. [11]	36, F	T3-6	NA	Meta (NA)	Chemo/radio	D	2500	880	En-bloc	Thoracotomy post	Meningocele	E	18		NED

*Loca* location, *OML* other metastatic lesions, *Pri* primary, *LR* local recurrence, *Meta* metastasis, *PS* primary site, *AT* adjuvant therapy, *F-S Pre* preoperative Frankel score, *Appro* approach, *Comp* complication, *F-S post* postoperative Frankel score, *M* male, *F* female, *NA* not available, *Targ* targeted therapy, *Chemo* chemotherapy, *Radio* radiotherapy, *DOD* died of disease, *NED* no evidence of disease, *AWD* alive with disease

### Treatment details

All patients were surgically treated, where five of them also underwent adjuvant therapies such as chemotherapy, radiotherapy, and targeted therapy to manage their local and/or systematic diseases. All patients suffered from neurological defects due to cord and/or radical compression, and the majority number of patients had their symptoms alleviated postoperatively. Nine piecemeal and two en bloc surgeries were performed. The time of surgery and intraoperative blood loss varies, with an average of 301.1 ± 247.8 min (110–880) and 2162.5 ± 1395.9 ml (1000–5000), respectively. Reconstructions were performed for all patients postoperatively except for patient no.2 who underwent low sacrectomy.

Two patients suffered from postoperative complications. Patient no.5 was a 16-year-old girl, who developed wound disunion and necrosis after the first surgery. After conservative treatment for 20 days, she underwent a second surgical procedure where her wound healed 15 days thereafter. Moreover, one patient reported by Lizzati et al. suffered from meningocele after the en bloc resection but made a full recovery after 40 days of hospitalization.

### Follow-up

**Fig. 1:**
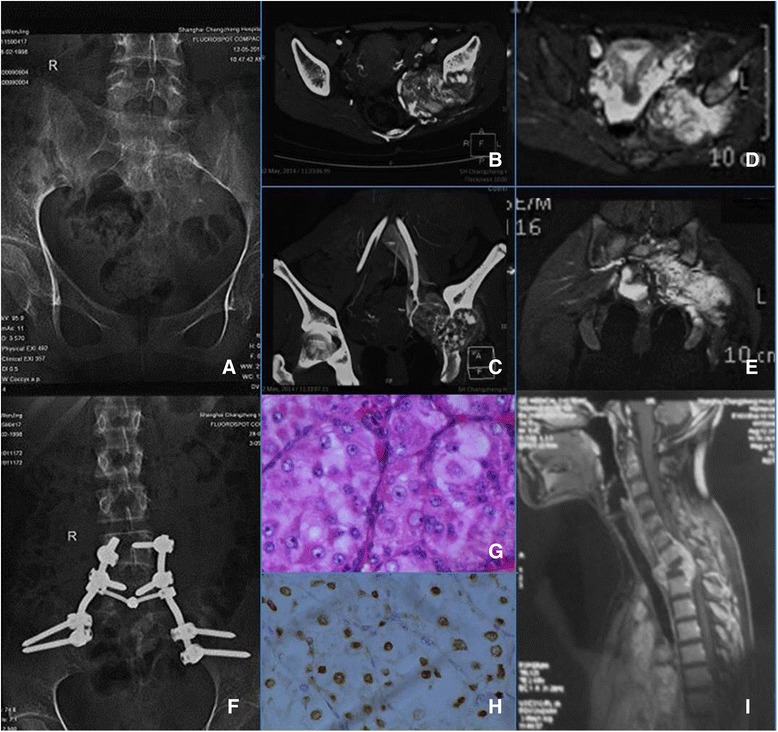
Patient no. 5 suffered from pain in the buttock and gait abnormality for 2 months, X-ray (**a**), CT (**b**, **c**), and MRI (**d**, **e**) were performed preoperatively and a large tumor in the sacrum was revealed. A fine needle biopsy confirmed ASPS where surgical resection was indicated. After the tumor resection, screw and rod reconstruction was performed (**f**). The post operative histological examination (**g**) and the TFE3 positive immunochemistry (**h**) confirmed the pathology diagnosis of ASPS. Metastasis was detected 3 months later (**i**) and two further surgeries were performed. Unfortunately, this patient succumbed to the disease 5 months after the first surgery

The mean follow-up time was 19.7 ± 8.8 months (8–34). Two patients had tumor local recurrence postoperatively and underwent further surgical procedures. Patient no.4 was alive with disease 21 months after the second surgery, and patient no.5 succumbed to the disease 4 months after her third surgery (Fig. 1). The poor outcome of this 16-year-old girl might be owing to local disease recurrence and the early onset of distant metastasis. The rest three patients were still alive at the time of the follow-up, where two of them had no evidence of disease. The two patients underwent en bloc resections and were reported earlier had different outcomes. One died 26 months postoperatively while the other one was alive with no evidence of disease after 18 months follow-up.

## Discussion

ASPS is a malignant tumor, which accounts only 0.4–1% of all soft tissue sarcomas, with a higher incidence amongst patients between 15–35 years of age, but rare before age 5 and after age 50 years [1, 2, 13, 14]. The disease has obvious female predominance in the first three decades and reverses thereafter, the reported female to male ratio is approximately 2:1 and it is well reflected in this case series [13]. Although spinal involvements were extremely rare for ASPS, both primary and metastatic ASPS that erode the spinal column were represented in this report. In metastatic ASPS, more lesions were found in the mobile spines, whereas primary ASPS was more commonly seen in the sacrum.

The symptoms of spinal ASPS lack specificity. It may resemble neurological defects which initially present as pain and numbness of the innervated areas, followed by loss of mobility and loss of sensory receptions; furthermore, in the worst cases, this may progress into paralysis as seen in this case series as well as published literatures. Some patients also developed a soft palpable mass at the paraspinal areas of the buttocks. The radiological features of spinal ASPS are consistent with lesions at other locations but with characteristic osseous destruction plus a paraspinal soft tissue mass [8, 15]. Magnetic resonance (MR) images may assist in the diagnosis and differential diagnosis of this relatively rare disease by a combination of heterogeneous high signal on both T1- and T2-weighted images, with or without multiple intra- or extra-tumoral signal voids [16].

Pathological examinations, including histological and genetic analyses, have been treated as the gold standard diagnostic criteria and have an increased number of applications recently [1, 17, 18]. While Christopherson et al firstly reported and named ASPS as a distinctive and unrecognized soft tissue tumor, the histological features of ASPS were later described by pathologists [19–21]. The most important feature of ASPS is the characteristic ASPL/TFE3 fusion recognized using immunohistochemistry examinations, which is considered of great diagnostic value [22].

Surgical management indeed offer the best prognosis and should be considered the most important and the most effective treatment strategy for spinal ASPS as previously suggested [11]. At early stages, patients are asymptomatic; however, upon diagnosis, there have always been neurological defects and large size tumors identified. Surgical resections can directly remove the majority or the entirety of the tumor mass, which quickly lead to spinal cord or radicular decompression. This can alleviate suffering, improve the quality of life, or even prolong life expectancy. Although en bloc resection have been widely accepted and considered to be the best choice for soft tissue sarcomas, including ASPS, but it was not always achievable [9, 12], this is usually due to the large tumor size and the complexity of the local anatomical structures. Spinal ASPS is always accompanied with a large soft tissue mass, which might infiltrate into the soft paraspinal spaces and/or erode the surrounding vital nerve roots and vessels. Whether these structures can be sacrificed should be cautiously considered based on the general condition of the patient, which includes systematic disease progression, potential prognoses, and the preference of the patient. Although en bloc resection may prolong survival, the sacrifice of certain vital structures may compromise the quality of life for patients. All five patients in this review and one from the literature underwent piecemeal resection, whereas the other two from the literature underwent resection using an en bloc fashion. The duration of surgery and intraoperative blood loss ranges, from our experience, the spinal ASPS is rich in the vessels and it was challenging to deal with the intraoperative blood loss. Preoperative embolization of the tumor vessels might be helpful but no conclusion could be drawn yet. These features highlighted the difficulties in the surgical management of spinal ASPS.

Unfortunately, the value of adjuvant therapies for ASPS was limited. Four of our patients received adjuvant therapies. In three cases, this may have helped to control the systematic disease, but no impact was seen on the spinal lesions. In fact, chemotherapy and radiotherapy are more commonly considered as palliative strategies for hospice care [23, 24]. A number of ongoing clinical trials are focusing on the targeted therapies of ASPS, but no conclusions can be drawn yet [25–28].

Currently, the recognized prognostic factors of ASPS are age, tumor size, and metastases status, whereas the histological feature is thought to have no prognostic significance [1, 5, 29, 30]. Three patients of our cases together with one from literature had metastatic spinal ASPS and were all successfully treated. The prognoses of these patients can be optimistic. However, the primary spinal ASPS may indicate a poor prognosis seen from both of our cases and the literature. Two of the patients with primary ASPS died while the other one (no. 3) had a local recurrence. Furthermore, the poor prognosis of patient no.5 may be a combination of young age, large tumor size, rapid lesion progression, poor neurological status, early multi-organ metastasis, ineligibility to en bloc resection, and lack of effective adjuvant therapies. From our preliminary clinical experience and knowledge, it can be concluded that the poor prognostic factors for spinal ASPS may include primary spinal lesions, severe neurological defects, rapid lesion progression with early metastasis, and ineligibility to en bloc resection; on the other hand, patients with metastatic spinal lesions and well-controlled systematic disease may have a good prognosis. However, this conclusion requires further investigation using larger sample size due to the limitation of small patient number in the current study.

## Conclusions

In summary, this report has presented a case series of five spinal ASPS patients and systematically reviewed related literatures. It is concluded that surgical management is the most important and the most effective treatment strategy for spinal ASPS while adjuvant therapies had little effects. The prognostic factors for spinal ASPS include primary or metastatic lesions, neurological defects, disease progression, systematic conditions, and resection approaches. The limitation of this study is the small patient number; however, due to the rare nature of this tumor type, clinical trials with large number of patients and randomized designs may require multi-institutional cooperation.

## Abbreviations

ASPS: Alveolar soft part sarcoma
AWD: Alive with disease
DOD: Died of disease
MR: Magnetic resonance
NED: No evidence of disease
